# Supplementary material for: SHREC: A framework for advancing next-generation computational phenotyping with large language models
Source: PLOS Digit Health. 2026 Feb 13;5(2):e0001217. doi: 10.1371/journal.pdig.0001217 (PMC12904566; doi:10.1371/journal.pdig.0001217)
Supplement: S1 Appendix — The supplementary material file contains prompts, concept notes, the application of PHEONA (Evaluation of PHEnotyping for Observational Health Data) to the Apply Algorithm step, and details on code availability. (PDF) [file pdig.0001217.s001.pdf]

Supplementary Material

Table of Contents

1 Prompts **2**
1.1 Respiratory Therapy Classification Prompt . . . 2
1.2 Medication Classification Prompt . . . 5
1.3 Phenotyping Prompt . . . 6

2 Concept Notes **9**

3 Using PHEONA for Apply Algorithm Step **10**
3.1 Methods . . . 10
3.1.1 Assessment of Resource Requirements . . . 10
3.1.2 Assessment of Model Ability . . . 10
3.2 Results . . . 10
3.2.1 Assessment of Resource Requirements . . . 10
3.2.2 Assessment of Model Ability . . . 11

4 Code Availability **15**

1.0. Prompts

1.1.0. Respiratory Therapy Classification Prompt

INSTRUCTIONS:

1) INPUT: The input contains the source table name of the concept and the concept description. The input will be provided to you in the following format: <input>INPUT</input>. **DO NOT FABRICATE AN INPUT**, even if the input is vague or unclear.

<input>
{description}
</input>

2) OBJECTIVE:

2a) Respond to the questions delimited by the <output></output> tags. Provide your answer **exactly ** in the format specified between the <output></output> tags. Do **NOT** do any of the following:
- Modify the format of the questions or answers.
- Provide explanations or additional details beyond the format requested.
- Fabricate an input description or add information that is not present in the description, even if it is empty or unclear.

2b) When determining if the description matches any of the concept categories, consider the following:
- **Match Criteria**: The concept description either EXACTLY MATCHES or PARTIALLY MATCHES the definition, terms, and/or acronyms for each concept. For PARTIAL MATCHES, if the **OVERALL ** meaning of the description aligns with the concept, it is a match. If the concept relates to removal, absence, or discontinuation of any of the concept categories, it is a match to that concept.
- **Acronyms**: Verify that any acronyms are explicitly defined in the concept when considering matches. Do **NOT ASSUME** the meaning of acronyms.
- **Unclear or Unmatched Descriptions**: If the concept description is unclear or the content of the description does not match any of the listed terms or acronyms, it is not a match to any concept.
- **No Assumptions**: Do **not fabricate** additional information or assume meanings for vague, incomplete, or ambiguous descriptions. Only use the **exact terms** provided in the description.

3) CONCEPT CRITERIA: These are the concept categories to consider:

3a) **Concept 1: Invasive Mechanical Ventilation (IMV)**
- **Definition**: This involves a tube in the trachea (either an endotracheal tube placed through the mouth, or rarely the nose, OR a surgically placed tracheostomy tube) connected to a ventilator, delivering mechanical ventilation.
- **Terms and Acronyms**:
  - Terms: Endotracheal tube, tracheostomy tube, tracheostomy, trach tube, trach (either unspecified or specific to mechanical ventilation), ventilator, vent, intubated, intubation, extubation, invasive mechanical ventilation, continuous positive airway pressure, pressure support, assist control vent mode, continuous mandatory ventilation vent mode, synchronized intermittent mandatory ventilation vent mode, pressure regulated volume control vent mode, airway pressure release ventilation vent mode.
  - Acronyms: ETT or ET (endotracheal tube), IMV (invasive mechanical ventilation), CPAP (continuous positive airway pressure), PS (pressure support), AC (assist control vent mode), CMV (continuous mandatory ventilation vent mode), SIMV (synchronized intermittent mandatory ventilation vent mode), PRVC (pressure regulated volume control vent mode), APRV or Bi-level (airway pressure release ventilation vent mode) .

3b) **Concept 2: High-Flow Nasal Insufflation/Nasal Cannula (HFNI/HFNC)**
- **Definition**: Oxygen is delivered through a nasal cannula at a flow rate above 15 L/min, with adjustments for oxygen concentration and flow rate.
- **Terms and Acronyms**:
  - Terms: Nasal cannula, Vapotherm, Airvo, Optiflow, High flow nasal cannula, Nasal high flow, Heated and humidified high flow nasal oxygen, Heated and humidified high flow nasal cannula, High flow nasal insufflation, High flow nasal oxygen, High flow nasal cannula, High flow cannula, High velocity nasal insufflation (FDA designation for Vapotherm).
  - Acronyms: HHFNO (heated and humidified high flow nasal oxygen), HHFNC (heated and humidified high flow nasal cannula), HFNI (high flow nasal insufflation), HFNO (high flow nasal oxygen), HFNC (high flow nasal cannula), NC (nasal cannula).

3c) **Concept 3: Non-Invasive Positive Pressure Ventilation (NIPPV)**
- **Definition**: Non-invasive ventilation via a facemask, where the clinician adjusts inspiratory pressure (the pressure that supports the breath), FiO2, and PEEP (the pressure maintained in the system at the end of a breath to keep the lungs from collapsing) to assist breathing.
- **Terms and Acronyms**:
  - Terms: Mask, mask ventilation, non-invasive ventilation, bi-level positive airway pressure, continuous positive airway pressure, inspiratory positive airway pressure, expiratory positive airway pressure, pressure support, average volume assured pressure support.
  - Acronyms: BiPAP (bilevel positive airway pressure), CPAP (continuous positive airway pressure), IPAP (inspiratory positive airway pressure), EPAP (expiratory positive airway pressure), PS (pressure support), AVAPS (average volume assured pressure support, an advanced mode that is exactly mechanical ventilation without the endotracheal tube), NIV (non-invasive ventilation).

3d) **Concept 4**: Convensional Oxygen Therapies
- **Definition**: Includes trach collar, venturi mask, trach tent, oximizer, oxymizer, oxymask, oximizer nasal cannula, misty ox, oxymask, oxi-mask, partial rebreather, simple mask.
- **Terms and Acronyms**:
  - Terms: Trach collar, venturi mask, trach tent, oximizer, oxymizer, oxymask, oximizer nasal cannula, misty ox, oxymask, oxi-mask, partial rebreather, simple mask.

3e) **Concept 5**: Settings for Mechanical Ventilation
- **Definition**: Includes settings for mechanical ventilation, such as FiO2, PEEP, respiratory rate, and Tidal Volume.
- **Terms and Acronyms**:
  - Terms: FiO2, PEEP, respiratory rate, tidal volume (TV).

3f) **Concept 6**: Respiratory Measurements
- **Definition**: Includes respiratory measurements, such as PaO2, SpO2, and pH.
- **Terms and Acronyms**:
  - Terms: PaO2, SpO2, pH, respiratory rate.

OUTPUT:
<output>
Q1) What was the exact provided input description? If the input description is empty or contains a medication, skip to Q5 and respond with 'NO'.
A1)
Q2) Think about whether the description is relevant to the definition, terms, or acronyms **ONLY FOR** Concept 1 - Invasive Mechanical Ventilation (IMV). Provide a brief judgment (1-2 sentences) and 'YES' or 'NO' for whether the description matches the concept. If 'YES', skip to Q5.
A2)

```
Q3) Think about whether the description is relevant to the definition, terms, or acronyms **ONLY
    FOR** Concept 2 - High-Flow Nasal Insufflation/Nasal Cannula (HFNI/HFNC). Provide a brief
    judgment (1-2 sentences) and 'YES' or 'NO' for whether the description matches the concept. If
    'YES', skip to Q5.
A3)
Q4) Think about whether the description is relevant to the definition, terms, or acronyms **ONLY
    FOR** Concept 3 - Non-Invasive Positive Pressure Ventilation (NIPPV). Provide a brief judgment
    (1-2 sentences) and 'YES' or 'NO' for whether the description matches the concept.
A4)
Q5) Based on your answers to the **previous three** questions (Q2-Q4), if the answer to any of the
    questions is 'YES', respond with 'YES' between the <answer></answer> tags. Otherwise, respond
    with 'NO' between the <answer></answer> tags.
A5) <answer>ANSWER</answer>
</output>
```

1.2.0. Medication Classification Prompt

```
INSTRUCTIONS:
1) INPUT: The input contains the source table name of the concept and the concept description. It may be incomplete or ambiguous. The input will be provided to you in the following format: <input>INPUT</input>. **DO NOT FABRICATE AN INPUT**, even if the description is vague or unclear.

<input>
{description}
</input>

2) OBJECTIVE:
2a) Respond to the questions delimited by the <output></output> tags. Provide your answer **exactly** in the format specified between the <output></output> tags. Do **NOT** do any of the following:
   - Modify the format of the questions or answers.
   - Provide explanations or additional details beyond the format requested.
   - Fabricate an input description or add information that is not present in the description, even if it is empty or unclear.
   - Use anything other than the exact terms provided in the description when responding to the questions.

2b) When determining if the description matches any of the listed medications, consider the following:
   - **Match Criteria**: The concept description contains an EXACT match to one of the medications listed in the question. Generic mentions of medication classes or other medications not listed in the question are **NOT VALID** matches.

OUTPUT:
<output>
Q1) What was the exact provided input description? If there is no medication listed, skip to Q4 and respond with 'NO'.
A1)
Q2) Think through the input description. Does the description match one of these specific sedatives: Etomidate, Ketamine, Midazolam (Versed), Propofol, Dexmedetomidine (Precedex), Fentanyl, Morphine, Hydromorphone (Dilaudid), Thiopental, or Cisatracurium? Provide YES/NO with a brief judgment, no more than one sentence.
A2)
Q3) Think through the input description. Does the description match one of these specific paralytics: Rocuronium, Succinylcholine, or Vecuronium? Provide YES/NO with a brief judgment, no more than one sentence.
A3)
Q4) Based on your answers to the **previous two** questions (Q2-Q3), if the answer to either question is 'YES', respond with 'YES' between the <answer></answer> tags. Otherwise, respond with 'NO' between the <answer></answer> tags.
A4) <answer>ANSWER</answer>
</output>
```

1.3.0. Phenotyping Prompt

```
INSTRUCTIONS:
1) INPUT: The input, delimited by <input></input>, will contain a SERIES OF RECORDS from a patient'
    s stay in the ICU. Each individual record (or row) will contain a description and will be
    ordered based on the occurrence of the description in the patient's stay. Each record will be
    in the following format: ORDER OF RECORD: <description>. **DO NOT** fabricate any information
    or make assumptions about the patient's records.

<input>
{description}
</input>

2) OBJECTIVE: Respond to the questions delimited by the <output></output> tags, including the
    delimiters in your response. Provide your answer **exactly** in the format specified between
    the <output></output> tags. Do **NOT** do any of the following:
   - Modify the format of the questions or answers.
   - Provide explanations or additional details beyond the format requested.
   - Fabricate an input or add information that is not present in the input, even if it is empty
      or unclear.

3) TREATMENTS:

   - **Treatment 1: Invasive Mechanical Ventilation (IMV)**
      - **INCLUSION CRITERIA**:
         1) At least ONE INDIVIDUAL record indicating the patient received **AT LEAST ONE** of
             the following medications: Specific Sedatives (Etomidate, Ketamine, Midazolam (
             Versed), Propofol, Dexmedetomidine (Precedex), Fentanyl, Morphine, Hydromorphone (
             Dilaudid), Thiopental, Cisatracurium) or Specific Paralytics (Rocuronium,
             Succinylcholine, Vecuronium).
         AND
         2) At least TWO INDIVIDUAL records indicating the patient was on invasive mechanical
             ventilation (IMV) or intubated. **EXCLUDES** records defining ventilation settings.
             Invasive mechanical ventilation involves a tube in the trachea (either an
             endotracheal tube placed through the mouth, or rarely the nose, OR a surgically
             placed tracheostomy tube) connected to a ventilator, delivering mechanical
             ventilation. Records with the following terms or acronyms should be considered for
             IMV unless otherwise indicated: ventilator, ETT or ET (endotracheal tube, trach tube
             ), tracheostomy/trach, PS (pressure support), AC (assist control vent mode), CMV (
             continuous mandatory ventilation vent mode), SIMV (synchronized intermittent
             mandatory ventilation vent mode), PRVC (pressure regulated volume control vent mode)
             , APRV or Bi-level (airway pressure release ventilation vent mode).

   - **Treatment 2: Non-Invasive Positive Pressure Ventilation (NIPPV)**
      - **INCLUSION CRITERIA**:
         1) At least TWO INDIVIDUAL records indicating the patient was on non-invasive positive
             pressure ventilation (NIPPV) **THAT DOES NOT INDICATE** high flow nasal insufflation
             /cannula or nasal cannula. Also **EXCLUDES** records defining ventilation settings.
             Non-invasive positive pressure ventilation involves ventilation via a facemask,
             where the clinician adjusts pressure and oxygen settings. Records with the following
              terms and acronyms should be considered NIPPV unless otherwise indicated: mask,
             mask ventilation, NIV (non-invasive ventilation), BiPAP (bilevel positive airway
             pressure), CPAP (continuous positive airway pressure), IPAP (inspiratory positive
             airway pressure), EPAP (expiratory positive airway pressure), AVAPS (average volume
             assured pressure support).

   - **Treatment 3: High-Flow Nasal Insufflation/Nasal Cannula (HFNI/HFNC) or Nasal Cannula**
      - **INCLUSION CRITERIA**:
         1) The criteria for NIPPV is met where the records are **INDEPENDENT** of any records
```

indicating HFNI or nasal cannula.

AND

2) At least ONE INDIVIDUAL record indicating the patient was on high flow nasal insufflation/cannula or nasal cannula. HFNI/HFNC involves oxygen delivery through a nasal cannula at a flow rate above 15 L/min, with adjustments for oxygen concentration and flow rate. Records with the following terms and acronyms should be considered HFNI/HFNC unless otherwise indicated: nasal cannula (NC), high flow nasal cannula, high flow nasal oxygen, high flow nasal insufflation, high flow nasal therapy, high flow nasal oxygen therapy, high flow nasal oxygen delivery, high flow nasal oxygen therapy (HFNOT), Optiflow, Vapotherm, Airvo.

OUTPUT:
<output>
SUMMARY:
Q1) Summarize the input records in 3-5 sentences.
A1)

TREATMENT TYPES:
Q2) Are any of the required medications present? If so, are there at least TWO INDIVIDUAL records indicating the patient was on invasive mechanical ventilation (IMV) or intubated? Provide a brief judgment (1-2 sentences) and 'YES' or 'NO' for whether the inclusion criteria for IMV is met.
A2)

Q3) Are there at least TWO INDEPENDENT records indicating the patient was on NIPPV that are ALSO INDEPENDENT of any records indicating HFNI or nasal cannula? Provide a brief judgment (1-2 sentences) and 'YES' or 'NO' for whether the inclusion criteria for NIPPV is met.
A3)

Q4) Based on the records provided, was the criteria for NIPPV met first? If the criteria for NIPPV was not met, then the criteria for HFNI is also not met. If the criteria for NIPPV was met, is there at least ONE ADDITIONAL record indicating HFNI or nasal cannula? Provide a brief judgment (1-2 sentences) and 'YES' or 'NO' for whether the inclusion criteria for HFNI is met.
A4)

TREATMENT ORDERING:
Q5) Based on the previous three questions (Q2-Q4), was there MORE THAN ONE treatment present? **REMEMBER**: If the criteria for HFNI is met, then ONLY HFNI applies, **NOT** NIPPV or HFNI and NIPPV. If so, list the treatments and skip to Q6. If not, skip to Q8.
A5)

Q6) What was the start and end record orders for each of the following: 1) IMV, 2) NIPPV (if applicable), and 3) HFNI (if applicable)? Provide a brief judgment (1-2 sentences).
A6)

Q7) Based on the start and end record orders, are the NIPPV or HFNI records independent of the IMV records? In other words, were ALL of the QUALIFYING RECORDS for NIPPV or HFNI completely BEFORE or AFTER the IMV records and NOT BETWEEN the IMV records? Provide a brief judgment (1-2 sentences). Remember, the QUALIFYING RECORDS for HFNI include the qualifying NIPPV records and an additional record indicating HFNI or nasal cannula so if HFNI was present, ALL THE QUALIFYING RECORDS must be independent of the IMV records.
A7)

FINAL CLASSIFICATION:
Q8) Based on your answers to the previous questions (Q2-Q7), which category does the patient's records fall under? **ONLY** respond with **ONE** of the following: IMV ONLY, NIPPV ONLY, HFNI ONLY, NIPPV TO IMV, HFNI TO IMV, IMV TO NIPPV, IMV TO HFNI, or NONE (if no records or specific treatments were present).
A8)

```
</output>
```

2.0. Concept Notes

Table 1: Concept notes produced by two clinician experts for each of the concepts of interest for phenotyping: Invasive Mechanical Ventilation (IMV), Noninvasive Positive Pressure Ventilation (NIPPV), High-Flow Nasal Insufflation (HFNI), and specific medications for IMV.

**Concept**	**Clinical Definition**	**Related Keywords and Acronyms**	**Common Recording Practices, Issues, or Discrepancies**
Invasive Mechanical Ventilation (IMV)	Tube in the trachea – e.g. “endotracheal tube” placed via mouth/orally or (rarely nose/nasally), or surgical tracheostomy tube – that connects to a ventilator. Intubation usually has a procedure note. Extubation may lack one — absence of vent settings is key.	Vent, Ventilator, Intubated, ETT (endotracheal tube), CPAP, PS, AC, CMV, SIMV, PRVC, APRV/Bi-level, RR, FiO2 or O2%, PEEP, TV, Inspiratory pressure, T-hi, T-lo, P-hi, P-lo	Bolus drugs: Etomidate, Ketamine, Midazolam, Propofol, Rocuronium, Succinylcholine. Sedation: Propofol (infusion), Dexmedetomidine/Precedex, Midazolam, Fentanyl, Morphine, Dilaudid. RT flow sheets often document endotracheal tube depth and size.
Noninvasive Positive Pressure Ventilation (NIPPV)	Noninvasive breathing via facemask or helmet. Clinician sets inspiratory pressure, PEEP, FiO2, RR. Patient must be alert and breathing independently.	Mask, NIPPV, RR, FiO2, PEEP, PS, AVAPS, BIPAP, CPAP	Settings can mimic vent settings. Need corroborating info: lack of ETT, no paralytics, no intubation procedure.
High-Flow Nasal Insufflation (HFNI)	Heated, humidified high-flow oxygen via nasal cannula. Flow rates typically 15–40 L/min. Devices include Airvo (up to 70 L/min) and Vapotherm (up to 40 L/min).	Vapotherm, High flow cannula, HFNC, FiO2, Flow rate (L/min)	”High flow” may refer to different tech in EMRs. Vapotherm allows FiO2 and flow to be set independently. Terminology varies widely.
Specific Medications for IMV	Typically only used in intubated patients. Sedatives: Propofol, Dexmedetomidine, Midazolam (if infused). Paralytics: Cisatracurium, Rocuronium, Succinylcholine, Vecuronium.	Abbreviations: prop (Propofol), cist (Cisatracurium), etc.	Same considerations as IMV. Paralytics imply intubation. Precedex and ketamine can be used without a ventilator, but paralytics cannot.

3.0. Using PHEONA for Apply Algorithm Step

In this section, we describe the application of PHEONA (Evaluation of PHEnotyping for Observational Health Data), an evaluation framework for Large Language Model (LLM)-based methods for computational phenotyping.[1] Previously, we applied PHEONA to the task of *Concept Selection*.[1] In this study, we apply PHEONA to the *Apply Algorithm* step of SHREC (SHifting to language model-based REal-world Computational phenotyping) to determine the best model(s) for phenotyping.

3.1.0. Methods

3.1.1.0. Assessment of Resource Requirements

The resource requirements were assessed by comparing manual development of the computational phenotyping algorithm to automated LLM phenotyping. In the original study, manual development was performed over 6 months out of the total 18 months required for phenotype development. Development effort and schedule for the LLM-based approach was measured during prompt engineering for the initial sample of 100 constructed descriptions.

3.1.2.0. Assessment of Model Ability

To fully demonstrate the framework, we evaluated all components of PHEONA although we focused on response correctness, response latency, and response format accuracy for final evaluation of the appropriate model(s). We prioritized response correctness to ensure accuracy of the responses. Response latency and format accuracy were secondary evaluation metrics to ensure the selected model could quickly and consistently phenotype the entire cohort. The time to minimal viable prompt was measured on an initial sample of 100 constructed descriptions and the remaining metrics were measured using another random sample of 100 constructed descriptions to avoid data leakage. Similar to when we applied PHEONA for *Concept Selection*, we evaluated the phenotyping task using random samples to mimic the phenotyping process where ground truths are unlikely to be available early in the phenotyping process.

For prompt factors, the time to minimum viable prompt was measured as the time (in hours) for prompt engineering of the prompts for classification of the sample constructed descriptions for each model. Model response latency was calculated by averaging the response latencies across the 100 samples. We did not consider testing at multiple times during the day since we used internal environments for hosting the LLMs. For the model consistency component, the response format accuracy was determined by counting the number of responses returned in the exact format specified in the prompt across all constructed descriptions. We then tested the response consistency and prompt stability by first randomly selecting 10 constructed descriptions from the sample of 100. For response consistency, we generated 10 model results per constructed description and evaluated how many times the model returned the same phenotype. The prompt stability was tested by rearranging the phenotyping prompt and evaluating whether the model returned the same answer for each constructed description. The changes made to the prompt were the following: 1) Moved the objective to after the phenotype descriptions; 2) Reversed the ordering of questions within each sub-sections of questions in the prompt; and 3) Reversed the ordering of the phenotype descriptions. Each change was independently tested 10 times on each of the 10 sampled constructed descriptions for a total of 100 responses per change.

For response correctness, the accuracy was measured using the area under the receiver operating characteristic curve (AUROC) for each of the phenotypes. The quasi-accuracy was also measured with AUROC for each phenotype after reviewing all constructed descriptions and updating the ground truths based on the prompt logic to to avoid penalizing the LLM for errors from *Concept Selection*. Hallucination frequency was determined by assessing the number of factually incorrect statements, such as incorrect acronym interpretations or fabrication of records in the encounter. The severity of each hallucination was evaluated as follows: Minor (little to no impact on the model reasoning or final decision); Major (impact on model reasoning but not the final decision, i.e., the right decision made for the wrong reason); Critical (impact on both model reasoning and final decision).

3.2.0. Results

3.2.1.0. Assessment of Resource Requirements

The resource requirements comparison for both methods is presented in Table 2. From this comparison, we note that while the LLM-based methods required more specialized hardware and software, the time savings

for phenotyping was estimated to be significant when compared to manual development of the algorithm in the traditional approach, especially when considering that the actual phenotyping is performed automatically by the LLM. Therefore, we decided LLM-based methods would be appropriate to explore for phenotyping.

3.2.2.0. Assessment of Model Ability

For time to minimum viable prompt, since a prompt developed on one model was effective for the remaining models, we did not estimate any differences in this metric between models. For the response correctness metrics, there were no hallucinations detected for Gemma and Phi and there were two minor hallucinations detected for Mistral (Table 3). The results for the remaining metrics are presented in Table 4. Gemma underperformed with respect to model response latency (32.4 seconds compared to 23.4 seconds and faster), response format accuracy (26% compared to 100%), and accuracy (0.490 to 0.620 AUROC compared to 0.900 and higher) and thus, was not used for phenotyping the entire dataset. Results were varied for quasi-accuracy with all models under-performing on at least one phenotype. In general, performance on IMV Only improved for Mistral (AUROC of 0.900 to 0.970) and Phi (AUROC of 0.900 to 0.960) while performance for HFNI Only was low for all models (AUROC of 0.490 to 0.740).

References

[1] Sarah Pungitore, Shashank Yadav, and Vignesh Subbian. “PHEONA: An Evaluation Framework for Large Language Model-based Approaches to Computational Phenotyping”. In: *arXiv* arXiv:2503.19265 (Mar. 25, 2025). DOI: 10.48550/arXiv.2503.19265.

Table 2: Demonstration of the resource requirements to decide between a Large Language Model (LLM)-based or a traditional phenotyping approach for the task of phenotyping.

**Development Effort**			
**Criterion**	**Traditional Approach**	**LLM-based Approach**	**Verdict**
Model Host Environments	Due to PHI concerns, only internal environments were available for use.	Due to PHI concerns, only internal environments were available for use. Environments with GPU resources were available but restricted based on demand.	Traditional approach was superior due to ability to run model on multiple internal environments rather than being restricted to those with GPU access.
Hardware Requirements	No additional computational resources or cost expected.	Access to high performance research servers was limited based on demand but incurred no additional cost.	Traditional approach was marginally superior due to limits on GPU resources although neither approach incurred additional costs.
Software Requirements	No additional software requirements specific to phenotyping required.	Software specific to interacting with LLMs was required and troubleshooting was required to adapt software to high performance compute environment.	Traditional approach was superior due to extra setup time for LLM software.
**Development Schedule**			
**Criterion**	**Traditional Approach**	**LLM-based Approach**	**Verdict**
Time for Pipeline Development	An estimated 6 months of the total 18 months for ARF respiratory support therapies phenotype development were required for algorithm development.	Two weeks were required for prompt engineering on the initial sample of constructed descriptions. An estimated additional month would be required to gather all responses for all models based on sample latencies with models running in parallel.	LLM-based method was superior.
Time for Manual Review	Manual review of 5% of the phenotyped records was performed over 1 month.	No manual review was performed in this study, although we estimated 5% of records would require 1 month to review for validation.	No Advantage, since both methods required manual review of a sample to assess overall algorithm effectiveness.
Phenotype Runtime	The phenotyping runtime was 5 minutes for the entire dataset.	An single record would require an estimated 30 seconds to phenotype.	The traditional approach was superior since algorithm runtime, once developed, included phenotyping of all records.

Table 3: The constructed description, model response, and classification of the hallucination (Minor, Major, or Severe) detected for Mistral Small 24 billion. The hallucinated text of the model response is bolded.

Constructed Description	Model Response	Classification
1: MORPHINE INJ<br>2: 2 ML VIAL : MIDAZOLAM HCL 2 MG/2ML IJ SOLN<br>3: 2 ML - FENTANYL CITRATE 0.05 MG/ML IJ SOLN<br>4: respFlowSettings: PEEP: 6<br>5: respFlowSettings: Pressure Support: 6<br>6: 100 ML NDC : DEXMEDETOMIDINE HCL IN NACL 400 MCG/100ML IV SOLN<br>7: FENTANYL STANDARD INFUSION 10 MCG/ML PREMIX<br>8: Value: Maximal assist<br>9: Airway: Intubated/oral ETT<br>10: Ventilation: Ventilated - rapid wean/extubation<br>11: Value: Moderate assist<br>12: Airway: Not intubated/normal airway<br>13: Value: Minimal assist	A1) THE PATIENT RECEIVED SEVERAL MEDICATIONS INCLUDING MORPHINE, MIDAZOLAM, AND **HIGH FLOW NASAL OXYGEN.** THE PATIENT WAS INTUBATED AS INDICATED BY THE PRESENCE OF IMV RELATED RECORDS. THERE ARE NO RECORDS INDICATING NIPPV OR HFNI.	Minor - The model later states that no High-Flow Nasal Insufflation (HFNI) records were present.
1: O2 Admin Device: vent<br>2: MIDAZOLAM HCL<br>3: pulmonary—ventilation and oxygenation—mechanical ventilation<br>4: Airway: Intubated/oral ETT<br>5: Ventilation: Ventilated - with daily extubation evaluation<br>6: respFlowSettings: PEEP: 5<br>7: Propofol 10 MG/ML Inj 10 MG/ML VIAL<br>8: Propofol (mcg/kg/min)<br>9: respFlowSettings: PEEP: 10<br>10: respFlowSettings: FiO2: 80<br>11: O2 Admin Device: ventilator<br>12: Airway: Not intubated/normal airway<br>13: O2 Admin Device: nasal cannula<br>14: pulmonary—radiologic procedures / bronchoscopy—endotracheal tube removal<br>15: pulmonary—ventilation and oxygenation—oxygen therapy (< 40%)—nasal cannula<br>16: HYDROMORPHONE HCL<br>17: respFlowSettings: PEEP: 0	A1) THE PATIENT'S RECORDS INDICATE A SERIES OF INTERVENTIONS INCLUDING THE USE OF MIDAZOLAM AND **FENTANYL**, INTUBATION WITH AN ENDOTRACHEAL TUBE, MECHANICAL VENTILATION, AND THE APPLICATION OF A NASAL CANNULA. THERE ARE ALSO MENTIONS OF SPECIFIC VENTILATOR SETTINGS SUCH AS PEEP AND FIO2 ADJUSTMENTS. THE RECORDS SUGGEST A TRANSITION FROM INVASIVE MECHANICAL VENTILATION TO NON-INVASIVE SUPPORT.	Minor - The model correctly identified additional medications used for phenotyping.

Table 4: Demonstration of the model ability criteria to determine the best Large Language Model (LLM) for phenotyping between Gemma2 27 billion, Mistral Small 24 billion, and Phi-4 14 billion. When included, the denominators indicate the number of LLM responses that were evaluated for each metric based on the specific experiment implemented. Only the phenotypes included in the sample were evaluated for *Response Correctness*.

**Prompt Factors**			
**Criterion**	**Gemma**	**Mistral**	**Phi**
Model Response Latency	32.4 seconds	23.4 seconds	17.8 seconds
**Model Consistency**			
**Criterion**	**Gemma**	**Mistral**	**Phi**
Response Format Accuracy	26/100 (26%)	100/100 (100%)	100/100 (100%)
Response Consistency	96/100 (96%)	100/100 (100%)	100/100 (100%)
Prompt Stability			
Instructions moved	98/100 (98%)	90/100 (90%)	100/100 (100%)
Questions reversed	80/100 (80%)	90/100 (90%)	100/100 (100%)
Concept order reversed	30/100 (30%)	90/100 (90%)	100/100 (100%)
**Response Correctness**			
**Criterion**	**Gemma**	**Mistral**	**Phi**
Accuracy (AUROC[a])			
IMV[b] Only	0.620	0.900	0.900
NIPPV[c] Only	0.490	1.000	0.990
None	0.600	0.930	0.920
Quasi-Accuracy (AUROC[a])			
IMV[b] Only	0.560	0.970	0.960
NIPPV[c] Only	0.490	0.990	0.990
HFNI[d] Only	0.490	0.740	0.490
IMV[b] to NIPPV[c]	0.490	0.500	1.000
None	0.630	0.960	0.950

[a] AUROC: Area under the receiver operating characteristic curve.

[b] IMV: Invasive Mechanical Ventilation.

[c] NIPPV: Noninvasive Positive Pressure Ventilation.

[d] HFNI: High-Flow Nasal Insufflation.

4.0. Code Availability

All code was run with Python version 3.13.3. The code and requirements for this work can be found at: https://github.com/spungit/SHRECandPHEONA
